# Supplementary material for: Animal behavior is central in shaping the realized diel light niche
Source: Commun Biol. 2022 Jun 8;5:562. doi: 10.1038/s42003-022-03472-z (PMC9177748; doi:10.1038/s42003-022-03472-z)
Supplement: Supplementary file 2 — Supplementary Information [file 42003_2022_3472_MOESM2_ESM.pdf]

Supplementary Figures: 'Animal behavior is central in shaping the realized diel light niche'

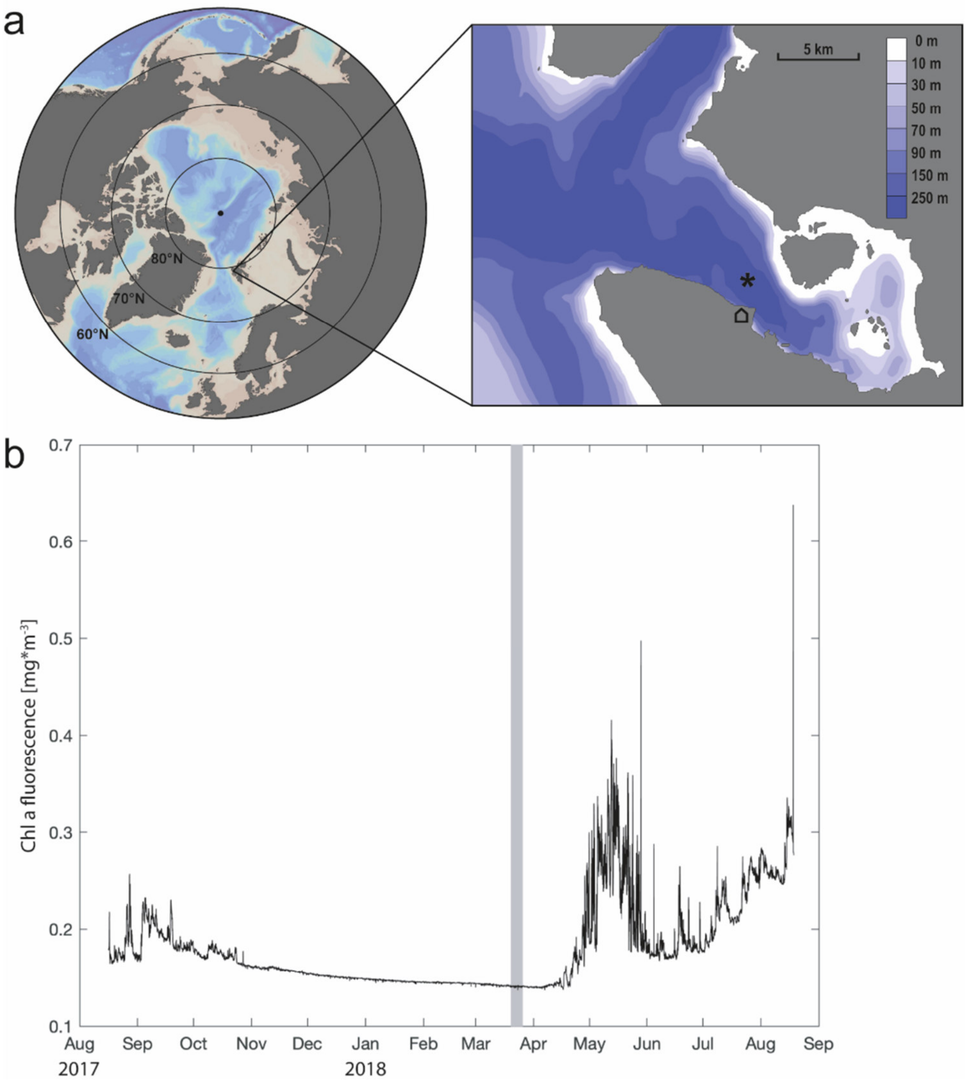

Supplementary Fig. 1: Study site characteristics. (a) Map of the Arctic and Kongsfjorden in the Svalbard archipelago. ***** indicates the site of the mooring (~230 m bottom depth). ⌂ indicates the location of the light sensor. (b) Chlorophyll a fluorescence (a proxy of phytoplankton concentration) at 33 m depth in Kongsfjorden in 2017/2018. The gray bar indicates the study period around the spring equinox.

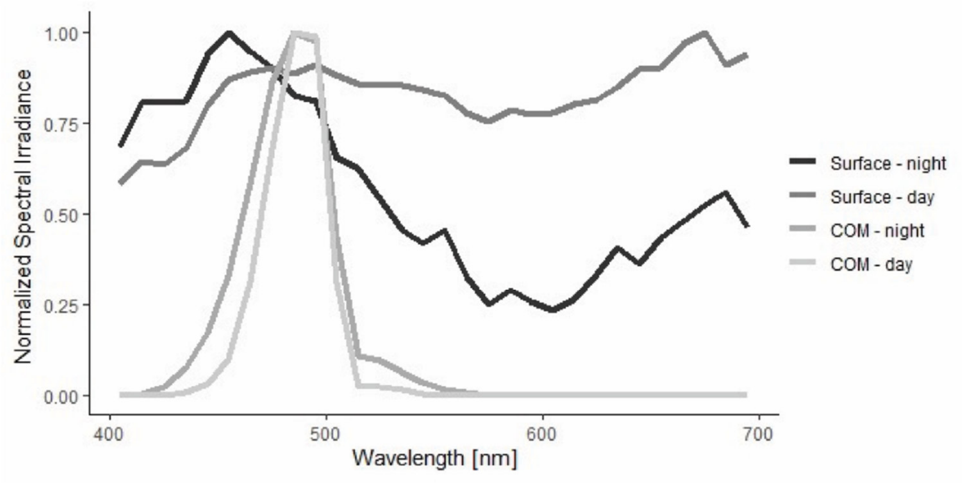

Supplementary Fig. 2: Relative spectral intensities. Spectral intensities on March 17th at the surface and realized at the zooplankton center of mass (COM) are shown for midday and midnight. Values were normalized separately, meaning that intensities are not comparable between surface and COM.

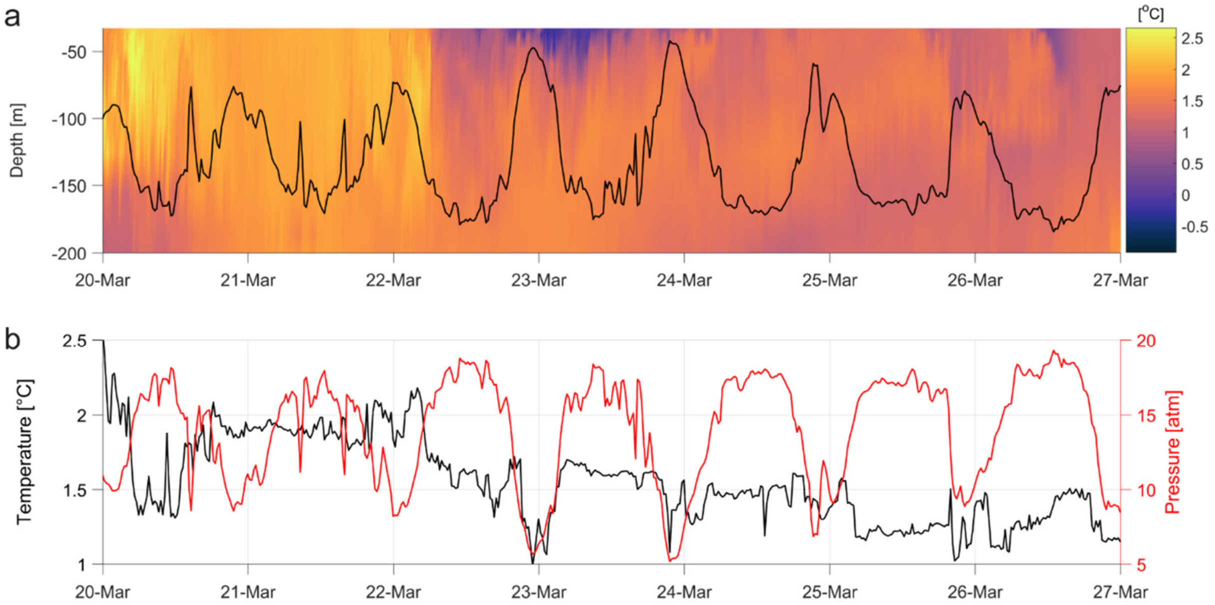

Supplementary Fig. 3: Realized temperature and pressure. (a) Temperature depth profile with zooplankton center of mass (COM, black line) added for illustration. (b) Realized diel cycles of temperature (black) and pressure (red) experienced at the zooplankton COM. Dates on the x-axis indicate the start (midnight) of the respective days. Lomb-Scargle analysis showed no significant rhythmicity for temperature at the COM (% rhythm = 0.4%, $p=0.36$), while pressure at the COM was significantly rhythmic with a 23.9h period (% rhythm = 75.0%, $p<0.001$).
