# Supplementary material for: Animal behavior is central in shaping the realized diel light niche
Source: Commun Biol. 2022 Jun 8;5:562. doi: 10.1038/s42003-022-03472-z (PMC9177748; doi:10.1038/s42003-022-03472-z)
Supplement: Supplementary file 4 — Reporting Summary [file 42003_2022_3472_MOESM4_ESM.pdf]

nature research

Corresponding author(s): Sören Häfker
soeren.haefker@univie.ac.at

Last updated by author(s): Apr 11, 2022

Reporting Summary

Nature Research wishes to improve the reproducibility of the work that we publish. This form provides structure for consistency and transparency in reporting. For further information on Nature Research policies, see our Editorial Policies and the Editorial Policy Checklist.

Statistics

For all statistical analyses, confirm that the following items are present in the figure legend, table legend, main text, or Methods section.

n/a	Confirmed	
☒	☐	The exact sample size (*n*) for each experimental group/condition, given as a discrete number and unit of measurement
☒	☐	A statement on whether measurements were taken from distinct samples or whether the same sample was measured repeatedly
☐	☒	The statistical test(s) used AND whether they are one- or two-sided<br>*Only common tests should be described solely by name; describe more complex techniques in the Methods section.*
☒	☐	A description of all covariates tested
☒	☐	A description of any assumptions or corrections, such as tests of normality and adjustment for multiple comparisons
☒	☐	A full description of the statistical parameters including central tendency (e.g. means) or other basic estimates (e.g. regression coefficient) AND variation (e.g. standard deviation) or associated estimates of uncertainty (e.g. confidence intervals)
☒	☐	For null hypothesis testing, the test statistic (e.g. *F*, *t*, *r*) with confidence intervals, effect sizes, degrees of freedom and *P* value noted<br>*Give P values as exact values whenever suitable.*
☒	☐	For Bayesian analysis, information on the choice of priors and Markov chain Monte Carlo settings
☒	☐	For hierarchical and complex designs, identification of the appropriate level for tests and full reporting of outcomes
☒	☐	Estimates of effect sizes (e.g. Cohen's *d*, Pearson's *r*), indicating how they were calculated

Our web collection on statistics for biologists contains articles on many of the points above.

Software and code

Policy information about availability of computer code

Data collection	No software was used.
Data analysis	Hydrolight radiative transfer model (Sequoia Scientific), TSA Cosinor v6.3, RStudio v1.1.453, Adobe Illustrator v25.2.3

For manuscripts utilizing custom algorithms or software that are central to the research but not yet described in published literature, software must be made available to editors and reviewers. We strongly encourage code deposition in a community repository (e.g. GitHub). See the Nature Research guidelines for submitting code & software for further information.

Data

Policy information about availability of data

All manuscripts must include a data availability statement. This statement should provide the following information, where applicable:
- Accession codes, unique identifiers, or web links for publicly available datasets
- A list of figures that have associated raw data
- A description of any restrictions on data availability

Raw data associated with the figures is provided in a supplemental Excel-file. Any additional information can be obtained directly from the authors.

Field-specific reporting

Please select the one below that is the best fit for your research. If you are not sure, read the appropriate sections before making your selection.

☐ Life sciences ☐ Behavioural & social sciences ☒ Ecological, evolutionary & environmental sciences

For a reference copy of the document with all sections, see nature.com/documents/nr-reporting-summary-flat.pdf

Ecological, evolutionary & environmental sciences study design

All studies must disclose on these points even when the disclosure is negative.

Study description	Effects of zooplankton diel vertical migration on the realized environment were investigated by measuring zooplnakton distribution, light conditions and temperature in an Arctic fjord during the spring equinox.
Research sample	An Arctic marine zooplankton community was investigated.
Sampling strategy	n/a
Data collection	Data on zooplankton distribution and temperature were recorded by ADCPs and loggers attached to a mooring deployed in Kongsfjorden, Svalbard. Light conditions were recorded by a land-based spectral sensor.
Timing and spatial scale	Data originate from the 20th-26th of March 2018. ADCP and light data were recorded at 20 min intervals. Temeprature data were recorded at 12 min intervals. ADCP and temperature data cover a depth range of ~180 m. Depth changes in lihgt conditions were modelled.
Data exclusions	No data were excluded.
Reproducibility	A 7-day period was investigate to make sure that observed patterns are consistent and not associated with conditions on a specific day.
Randomization	n/a
Blinding	Blinding was not relevant as there was no comparison of different groups or treatments.

Did the study involve field work? ☒ Yes ☐ No

Field work, collection and transport

Field conditions	There were no major weather events (precipitations, storms) during the study period that could have affected the measured parameters.
Location	Data were recorded in Kongsfjorden, an Arctic fjord in the Svalbard archipelago (78° 57.54’ N, 11° 49.44’ E). Bottom depth at the study site was ~230 m.
Access & import/export	All work was carried out in compliance with local regulations. Aside from digital data, there was no import/export of sample material.
Disturbance	The study did not cause any disturbance to the environment. All deployed equipment was retrieved.

Reporting for specific materials, systems and methods

We require information from authors about some types of materials, experimental systems and methods used in many studies. Here, indicate whether each material, system or method listed is relevant to your study. If you are not sure if a list item applies to your research, read the appropriate section before selecting a response.

Materials & experimental systems

n/a	Involved in the study	
☒	☐	Antibodies
☒	☐	Eukaryotic cell lines
☒	☐	Palaeontology and archaeology
☐	☒	Animals and other organisms
☒	☐	Human research participants
☒	☐	Clinical data
☒	☐	Dual use research of concern

Methods

n/a	Involved in the study	
☒	☐	ChIP-seq
☒	☐	Flow cytometry
☒	☐	MRI-based neuroimaging

Animals and other organisms

Policy information about studies involving animals; ARRIVE guidelines recommended for reporting animal research

Laboratory animals	n/a
Wild animals	The investigated zooplankton community was dominated by Arctic krill (Thysanoessa spp.) and copepods (Calanus spp.). No animals were captured or killed.
Field-collected samples	The study did not involve any samples collected from the field.
Ethics oversight	n/a

Note that full information on the approval of the study protocol must also be provided in the manuscript.
