# Supplementary material for: Animal behavior is central in shaping the realized diel light niche
Source: Commun Biol. 2022 Jun 8;5:562. doi: 10.1038/s42003-022-03472-z (PMC9177748; doi:10.1038/s42003-022-03472-z)
Supplement: Supplementary file 5 — Description of Additional Supplementary Files [file 42003_2022_3472_MOESM5_ESM.pdf]

Description of Additional Supplementary Files

File name: Supplementary Data 1
Description: Source data behind the graphs in the paper.
